# Associations between the neuron-specific glucocorticoid receptor (NR3C1) Bcl-1 polymorphisms and suicide in cancer patients within the first year of diagnosis

**DOI:** 10.1186/s12993-016-0104-1

**Published:** 2016-07-11

**Authors:** Subin Park, Jin Pyo Hong, Jong-Keuk Lee, Young-Mi Park, Yangsoon Park, Juri Jeon, Myeong Hee Ahn, Se Chang Yoon

**Affiliations:** Research Planning Division, Mental Health Research Institute, National Center for Mental Health, Seoul, Republic of Korea; Department of Psychiatry, Samsung Medical Center, Sungkyunkwan University School of Medicine, 81 Irwon-Ro Gangnam-gu, Seoul, 135-710 Republic of Korea; Asan Institute for Life Sciences, University of Ulsan College of Medicine, Seoul, Republic of Korea; Department of Pathology, Asan Medical Center, University of Ulsan College of Medicine, Seoul, Republic of Korea; Department of Psychiatry, Asan Medical Center, University of Ulsan College of Medicine, Seoul, Republic of Korea

**Keywords:** Cancer, Suicide, NR3C1, Genetics

## Abstract

**Background:**

Cancer diagnosis is associated with an increased suicide risk, particularly within the first 1 year after diagnosis of cancer. Abnormal function of the hypothalamic–pituitary–adrenal axis has been implicated in the pathophysiology of depression and suicide. We examined genetic associations of the functional Bcl-1 polymorphism of (rs41423247) neuron-specific glucocorticoid receptor (NR3C1) gene, with death by suicide in cancer patients. Suicides occurring within a year of cancer diagnosis (‘early suicide’) were considered separately from those suicides during the second or subsequent year (‘late suicide’) after cancer diagnosis.

**Methods:**

The subjects consisted of 343 cancer patients admitted to a general hospital in Seoul, South Korea from 1996 to 2009, of which 182 had died by suicide and 161 were alive on December 31, 2009. Genomic DNA was extracted from formalin-fixed paraffin-embedded tissue sample of patients with cancer. We conducted a case-control association analysis of Bcl-1 polymorphism of NR3C1 gene.

**Results:**

Subjects carrying the GG genotype of Bcl-1 polymorphism were at increased risk of early suicide when compared to those carrying the CC genotype (OR 3.80, 95 % CI 1.02–14.16, *p* = .047). Similarly, those individuals carrying the GG genotype (recessive mode) had an increased risk of early suicide relative to the CC or CG genotype (OR 3.71, 95 % CI 1.03–13.43, *p* = .045). However, there were no differences in the genotype distributions of the NR3C1 Bcl-1 polymorphism between late suicide cases and controls.

**Conclusions:**

Our findings suggest that the NR3C1 Bcl-1 polymorphisms may be involved in the susceptibility to suicide within the first year after cancer diagnosis among cancer patients in Korean population.

## Background

Cancer diagnosis is associated with an increased suicide risk [1–6]. Suicide risk is the highest within the first 1 year after diagnosis of cancer [1, 2, 7–9]. There was a significant decrease in the relative suicide risk over decades, and 5 years after diagnosis, the risk should be about the same as for the general population [10]. This highest suicide risk in early period after cancer diagnosis may be associated with emotional distress induced by diagnosis of cancer, progressive and life-threatening illness [11, 12]. Based on diathesis-stress model of suicidal behavior [13], among patients who were exposed to major psychological stress (i.e. cancer diagnosis), some individuals may be more vulnerable than others to develop suicidal behavior, and there may be genetic and biological differences that may make someone more or less vulnerable to suicide. To our best knowledge, there has been no genetic association study of suicides in cancer patients.

Depression and stress are among the major risk factors for suicidal behavior. Abnormalities of the hypothalamic–pituitary–adrenal (HPA) axis have been consistently reported in patients with major depressive disorder (MDD) [14] and in individuals who completed suicide [15, 16]. Whereas about 50 % of patients with MDD have an abnormal dexamethasone suppression test (DST), almost all those MDD patients who eventually completed suicide had an abnormal DST [17]. This suggests a significant association between an abnormal HPA axis and suicide, independently of MDD. The cortisol stress response has been identified as one of the most prominent candidate suicide endophenotypes [15–17].

The dysregulation of HPA axis function observed in depression and suicide is believed to be in part due to a disturbed feedback inhibition by endogenous corticoids [14, 17–19]. In humans, cortisol serves as a negative feedback regulator of HPA axis by its action on two types of receptors, glucocorticoid (GR) and mineralocorticoid (MR) receptors in the brain and the pituitary [20, 21]. It is believed that this impaired HPA axis negative feedback in depression and suicide may be related to altered GR and/or MR function. The most widely studied of polymorphisms associated with GR receptor expression is the Bcl-1 restriction fragment length polymorphism of the nuclear receptor subfamily 3, group C, member 1 (NR3C1) gene, located on chromosome 5q31. A biallelic polymorphism (Bcl-1, C > G) was identified downstream of the exon 2–intron 2 junction of the NR3C1 gene. The polymorphism might affect processing of the primary GR transcripts and cause increased GR sensitivity [22, 23], which affects strength of inhibitory feedback within the HPA axis [24]. Bcl-1 high-function (G) allele carriers have been observed to display reduced cortisol responses following psychosocial stressors [25] and greater suppression of cortisol after dexamethasone administration [23]. Such findings lead to the suggestion that psychosocial stressors, such as a cancer diagnosis, might have more pronounced effects in homozygous carriers of the G allele. Consistent with this, increased methylation that would cause reduced hippocampal NR3C1 expression has been observed in victims of suicide with a history of childhood abuse [26].

In the present study, we assessed genetic associations of the functional Bcl-1 polymorphism (rs41423247) of NR3C1 gene, with death by suicide in cancer patients, in particular suicide within the first year of diagnosis. To the best of our knowledge, this is the first association study of suicides in cancer patients, a homogenous stress-exposure group.

## Methods

### Data sources

The sampling pool for this study consisted of 164,497 adult patients (age ≥20 years) who had been diagnosed with cancer at the University Hospital in Seoul, South Korea, between 1996 and 2009 according to the International Classification of Disease for Oncology tenth revision (ICD-10). This nationwide registry accounts for approximately 10 % of newly diagnosed cancer cases [27]. Data were censored either on the date of death or on December 31, 2009. Person-years were calculated for each patient, and information on patient status was obtained from the database of the National Statistical Office (NSO), which compiles all death notices in South Korea.

The cause of death was established by linking to the NSO database that was matched with the hospital records based on the unique national identification number assigned to each Korean citizen.

### Selection of cases and controls

Suicide death was defined by ICD-10 codes X60–X84 (intentional self-harm). Among the 164,497 patients with cancer, 373 had died by suicide by December 31, 2009. Among the 373 cancer patients who had died by suicide, 185 patients had tissue for genetic analysis. One control patient was randomly selected from the study cohort using the statistical analysis system (SAS) program (SAS Institute, Cary, NC, USA) and matched by sex, age (±3 years), and the time of diagnosis (±2 years) with each patient who had died by suicide. Information about demographic factors and anatomic site of cancer was obtained from electronic medical records by two medical doctors. Cancer sites were organized into seven diagnostic groups. These included stomach cancer (C16.x), colorectal cancer (C18.x–20.x), biliary-pancreatic cancer (C22.1, C23.9, and C24.x–25.x), breast cancer (C50.x), liver cancer (C22.0), lung cancer (C34.x), and cancers of other sites (i.e., diagnoses not corresponding to the previously mentioned codes). Suicides occurring within a year of cancer diagnosis (‘early suicide’) were considered separately from those suicides during the second or subsequent year (‘late suicide’) after cancer diagnosis.

### DNA extraction and genotyping

Genomic DNA was extracted from formalin-fixed paraffin-embedded (FFPE) tissue sample of patients with cancer using a QIAamp® DNA FFPE Tissue Kit and the QIAGEN Deparaffinization solution (Qiagen, Hilden, Germany) according to the manufacturer’s instructions. Approximately 50 ng of genomic DNA was used for TaqMan® SNP genotyping. Among 370 samples, 23 samples below .1 µg were excluded from genetic analyses. Finally, the remaining 347 samples were included in the analysis. We genotyped NR3C1 Bcl-1 polymorphism with a TaqMan® SNP Genotyping Assays on a 7900HT Real-Time PCR System (Applied Biosystems, Foster City, CA, USA). There were four missing genotypes and finally, the 343 samples were included in statistical analyses.

### Statistical analyses

The distribution of the general demographic and clinical features between early suicide cases, late suicide cases, and controls was evaluated by using the 1-way analysis of variance test and the χ^2^ test for continuous and categorical variables, respectively. Agreement with Hardy–Weinberg equilibrium (HWE) for each SNP was tested using a goodness-of-fit χ^2^ test. Genotype distribution for each polymorphism was in agreement with the expected values of the HWE (*p* > .05). To investigate the association between the evaluated genotypes and death by suicide, binary logistic regression analyses were performed. For the main analyses, 3 set of dichotomized outcomes were defined as the all suicide cases versus controls, the early suicide cases versus controls, and the late suicide cases versus controls. A 2-tailed *p* value of <.05 was considered statistically significant. Data analyses were performed using the Statistical Package for Social Sciences (Version 21.0 for Windows; SPSS, Inc., Chicago, IL, USA). Power calculation was performed using the Quanto (http://www.hydra.usc.edu/gxe/) software to compute the statistical power of our study.

## Results

In total, our sample consisted of 47 early suicide cases, 135 late suicide cases, and 161 controls. Demographic and clinical factors of early and late suicide cases and controls are summarized in Table 1. There were no significant differences in sex and age at diagnosis between groups. In all groups, the most common cancer diagnosis was stomach cancer. The frequencies of biliary-pancreatic cancer in early suicide were higher than in late suicide (χ^2^ = 5.03, *p* = .025). The frequencies of other types of cancer were significantly different between groups.

**Table 1:** Characteristics of the study population

	Controls (N = 161)	Early suicide (N = 47)	Late suicide (N = 135)	χ^2/^F	*p*
Sex				1.18	.554
Male	114 (70.8)	31 (66.0)	100 (74.1)		
Female	48 (29.3)	16 (34.0)	36 (26.5)		
Age at diagnosis, mean (SD)	59.55 (11.71)	60.02 (11.24)	59.19 (10.98)	.10	.904
Diagnosis				7.40	.830
Stomach	47 (29.2)	15 (31.9)	41 (30.4)		
Colorectal	33 (20.5)	7 (14.9)	32 (23.7)		
Biliary-pancreas	22 (13.7)	10 (21.3)	12 (8.9)		
Breast	16 (9.9)	3 (6.4)	12 (8.9)		
Liver	3 (1.8)	0	2 (1.5)		
Lung	5 (3.0)	1 (2.1)	4 (2.9)		
Others	35 (21.3)	11 (23.4)	32 (23.5)		
Days to death		177.79 (91.84)	1425.96 (925.30)	−9.22	<.001

The allele and genotype distributions of the NR3C1 Bcl-1 polymorphism among the cases and controls are presented in Table 2. The genotype distributions of rs41423247 (*p* = .438) were found to be in HWE. There was no significant difference in allele and genotype distribution frequencies of NR3C1 Bcl-1 polymorphism between all included suicide cases and controls. When we stratified suicide cases by the time of occurrence of suicides (within a year of cancer diagnosis or later), we found that subjects carrying the GG genotype of Bcl-1 polymorphism were at increased risk of early suicide when compared to those carrying the CC genotype (OR 3.80, 95 % CI 1.02–14.16, *p* = .047). Similarly, those individuals carrying the GG genotype (recessive mode) had an increased risk of early suicide relative to the CC or CG genotype (OR 3.71, 95 % CI 1.03–13.43, *p* = .045). However, there were no differences in the genotype distributions of the NR3C1 Bcl-1 polymorphism between late suicide cases and controls.

**Table 2:** Genotype distributions and allele frequencies of Bcl-1 polymorphism of NR3C1 gene between suicide cases and controls

	Controls (N = 161)	Suicide (N = 182)	Early suicide (N = 47)	Late suicide (N = 135)	Suicide vs. controls		Early suicide vs. controls		Late suicide vs. controls	
					OR (95 % CI)	*p*	OR (95 % CI)	*p*	OR (95 % CI)	*p*
Allele										
C	251	280	67	213	1		1		1	
G	71	84	27	57	1.06 (.74–1.52)	.748	1.43 (.85–2.39)	.181	.95 (.64–1.40)	.782
Codominant										
CC	95	108	25	83	1		1		1	
CG	61	64	17	47	.92 (.59–1.44)	.724	1.06 (.53–2.12)	.872	.88 (.55–1.43)	.609
GG	5	10	5	5	1.76 (.58–5.33)	.318	3.80 (1.02–14.16)	.047	1.15 (.32–4.09)	.835
Dominant										
CC	95	108	25	83	1		1		1	
CG + GG	66	74	22	52	.99 (.64–1.52)	.950	1.27 (.66–2.44)	.478	.90 (.57–1.44)	.665
Recessive										
CC + CG	156	172	42	130	1		1		1	
GG	5	10	5	5	1.81 (.61–5.42)	.287	3.71 (1.03–13.43)	.045	1.20 (.34–4.24)	.777

*NR3C1* neuron-specific glucocorticoid receptor, *OR* odd ratio, *CI* confidence interval

## Discussion

This is the first genetic association study on death by suicide of homogenous group exposed to severe psychological stressor, a cancer diagnosis. We found significant differences in genotypic and allelic frequencies of the NR3C1 Bcl-1 polymorphism between early suicide cases and non-suicide controls.

HPA-axis hyperactivity, assessed by means of the DST or 24 h urinary cortisol samples, has been associated with completed suicide among depressed patients. Several studies have reported that suicidal individuals or first-degree relatives of suicide decedents fail to mount a proper HPA axis response to stress, and the cortisol stress response has been identified as one of the most prominent candidate suicide endophenotypes [15–17]. NR3C1 Bcl-1 high function (G) allele carriers have been observed to display reduced cortisol responses following psychosocial stressors [28, 29] and greater suppression of cortisol after dexamethasone [23]. Such findings lead to the conjecture that traumatic experiences might have more pronounced effects in individuals whose genetic propensities code for lesser modulatory feedback within the HPA axis.

In the present study, relative to cancer patients with the CC genotype, those with the GG genotype of Bcl-1 polymorphism had a 3.8-fold higher risk of early suicides, although there were no significant differences in genotype or allele frequencies distribution between total suicide cases and controls. The psychological stress induced by the cancer diagnosis itself may give rise to early suicides, whereas the disease burden, fatigue, exhaustion, and aggravation of preexisting psychiatric disorder may be associated with late suicide [7]. Our results suggest that the Bcl-1 G allele, which reduces cortical response to psychosocial stressor (in this case, the cancer diagnosis), is related to early suicide in cancer patients.

Because of the limited availability of post-mortem brain tissues, there have been only a few studies examining protein and gene expression of GR in suicide completers [26, 30]. Pandey et al. [30] found that the mRNA and protein expression of GR is decreased in the prefrontal cortex and amygdale of teenage suicide victims compared with control subjects. In a postmortem brain study by McGowan et al. [26], increased methylation in the exon 1F NR3C1 promoter, that would cause reduced hippocampal NR3C1 expression, was observed in suicide completers with a history of childhood abuse. This result is consistent with our finding of contribution of NR3C1 gene in suicide completion of individuals exposed to severe psychological stressor.

A limitation of the study was the relatively small sample size. On calculating the statistical power of the study, we found that approximately 524 case samples are required to perform a matched case–control study with 80 % power to detect a relative risk of 2.0 in an estimated prevalence of suicide in cancer patients of 60/100,000 [1]. However, in our study, we used a smaller sample size (161 controls and 182 cases). That may be one of the reasons why we could not find any significance in the association between total suicide cases and controls. Furthermore, based on our results shown in Table 2, we found that our study has 65.4 % power with a relative risk of 3.71. As our study has limited power, further study is necessary in an independent sample set with a large sample size. Additionally, we evaluated only one SNP on the NR3C1 gene. Although the NR3C1 gene may affect suicides in cancer patients, the influence of, and interactions between, many target genes, should be considered. Finally, as this was a retrospective chart-review study, we could not evaluate psychiatric symptoms after a cancer diagnosis, which are known to have an important influence on suicide in cancer patients.

## Conclusions

Our findings suggest that the NR3C1 Bcl-1 polymorphisms may be involved in the susceptibility to suicide within the first year after cancer diagnosis among cancer patients in Korean population. The study must be replicated in studies using larger samples and with using more SNPs.

## Abbreviations

HPA: abnormal function of the hypothalamic–pituitary–adrenal
rs41423247: the functional Bcl-1 polymorphism
NR3C1: neuron-specific glucocorticoid receptor
MDD: major depressive disorder
